# Transudative pleural effusion in pleuritis associated with immunoglobulin G4‐related disease diagnosed by thoracoscopy under local anaesthesia

**DOI:** 10.1002/rcr2.1404

**Published:** 2024-06-17

**Authors:** Yuto Kato, Kentaro Fukunaga, Aya Ooka, Shunichi Tokuoka, Yoko Kataoka, Takuya Fujita, Hiroyuki Sugihara, Masafumi Yamaguchi

**Affiliations:** ^1^ Department of Respiratory Medicine Koka Public Hospital Koka Japan; ^2^ Department of Thoracic Surgery Koka Public Hospital Koka Japan; ^3^ Department of Diagnostic Pathology Koka Public Hospital Koka Japan; ^4^ Division of Respiratory Medicine, Department of Medicine Shiga University of Medical Science Otsu Japan

**Keywords:** IgG4‐related disease, pleuritis, thoracoscopy, transudative pleural effusion

## Abstract

Immunoglobulin G4 (IgG4)‐related disease is a chronic inflammatory condition often characterized by exudative pleural effusions. However, transudative pleural effusions, like in the presented case of an 80‐year‐old man with multiple comorbidities, are less common but possible. Despite initial treatment with diuretics, the effusion persisted, prompting further investigation. Medical thoracoscopy revealed lymphatic follicle hyperplasia and an abundance of IgG4‐positive plasmacytoid cells, confirming IgG4‐related pleuritis. This case underscores the importance of considering inflammatory etiologies, such as IgG4‐related disease, when faced with unresponsive transudative pleural effusions. Thoracoscopy serves as a valuable diagnostic tool in such scenarios, allowing for precise diagnosis and appropriate management.

## Graphical abstract

We report a unique case of transudative pleural effusion associated with IgG4‐related disease in an 80‐year‐old man with multiple comorbidities. Despite initial treatment with diuretics, the effusion persisted, prompting further investigation. Medical thoracoscopy revealed lymphatic follicle hyperplasia and an abundance of IgG4‐positive plasmacytoid cells, confirming IgG4‐related pleuritis.

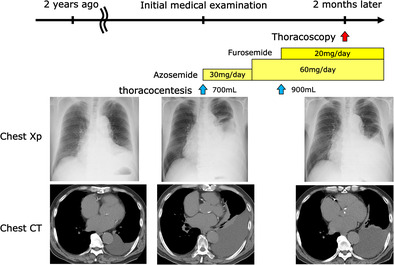

## INTRODUCTION

Immunoglobulin G4 (IgG4)‐related disease is a chronic systemic disorder affecting various organs. IgG4‐related pleuritis is rare, with around 20 reported cases. Typically, IgG4‐related pleural effusions are lymphocyte‐predominant exudative effusions with mild elevation of adenosine deaminase (ADA). Here, we report a unique case of transudative pleural effusion associated with IgG4‐related disease.

## CASE REPORT

An 80‐year‐old Japanese male, under the care of the cardiology department for conditions including arteriosclerosis obliterans, chronic atrial fibrillation, and diabetes mellitus, effectively manages these comorbidities through prescribed medication. A former smoker, he has no history of autoimmune diseases, multiple myeloma, or asbestos exposure, and he has not undergone tuberculosis treatment. Additionally, there is no family history of autoimmune disease or tuberculosis. Approximately 2 years ago, a CT scan identified a small left pleural effusion, which was placed under observation. A chest x‐ray revealed a notable increase in left pleural fluid, prompting referral to our department for further evaluation. While heart sounds were normal, decreased breath sounds were observed in the left lung field. The patient reported no spontaneous pain or tenderness in the chest. His abdomen exhibited a flat and soft appearance, without tenderness or abnormal bowel sounds. Pitting edema was absent in both forearms and legs. No motor or sensory deficits were identified in his extremities, and he denied experiencing arthralgia or morning stiffness associated with rheumatoid arthritis.

Inflammatory markers, such as C‐reactive protein (CRP) and tumour markers, were within the normal range. Autoantibodies yielded serologically negative results. Enzyme‐Linked Immunospot (ELISPOT) testing returned a positive result. Serum immunoglobulin G (IgG) measured at 2344 mg/dL, including 736 mg/dL for IgG4 (normal IgG range: 870–1700 mg/dL, normal IgG4 range: 4–108 mg/dL). Serum IgG4 is routinely performed to examine pleural effusion of unknown origin.

Chest x‐ray and CT scans revealed left pleural effusion without abnormal lung shadows or pleural thickening. A pleural puncture was performed, extracting 700 mL of pleural fluid. The left pleural effusion exhibited a yellow colour, primarily composed of lymphocytes, with a total protein concentration of 2.8 g/dL, a lactate dehydrogenase level of 62 IU/L, and an adenosine deaminase (ADA) level of 18.3 U/L. No microorganisms, including Mycobacterium tuberculosis, were detected in the pleural effusion. The presence of Class III (malignancy suspected) cells in the pleural effusion raises suspicion for malignant pleural mesothelioma.

**FIGURE 1:**
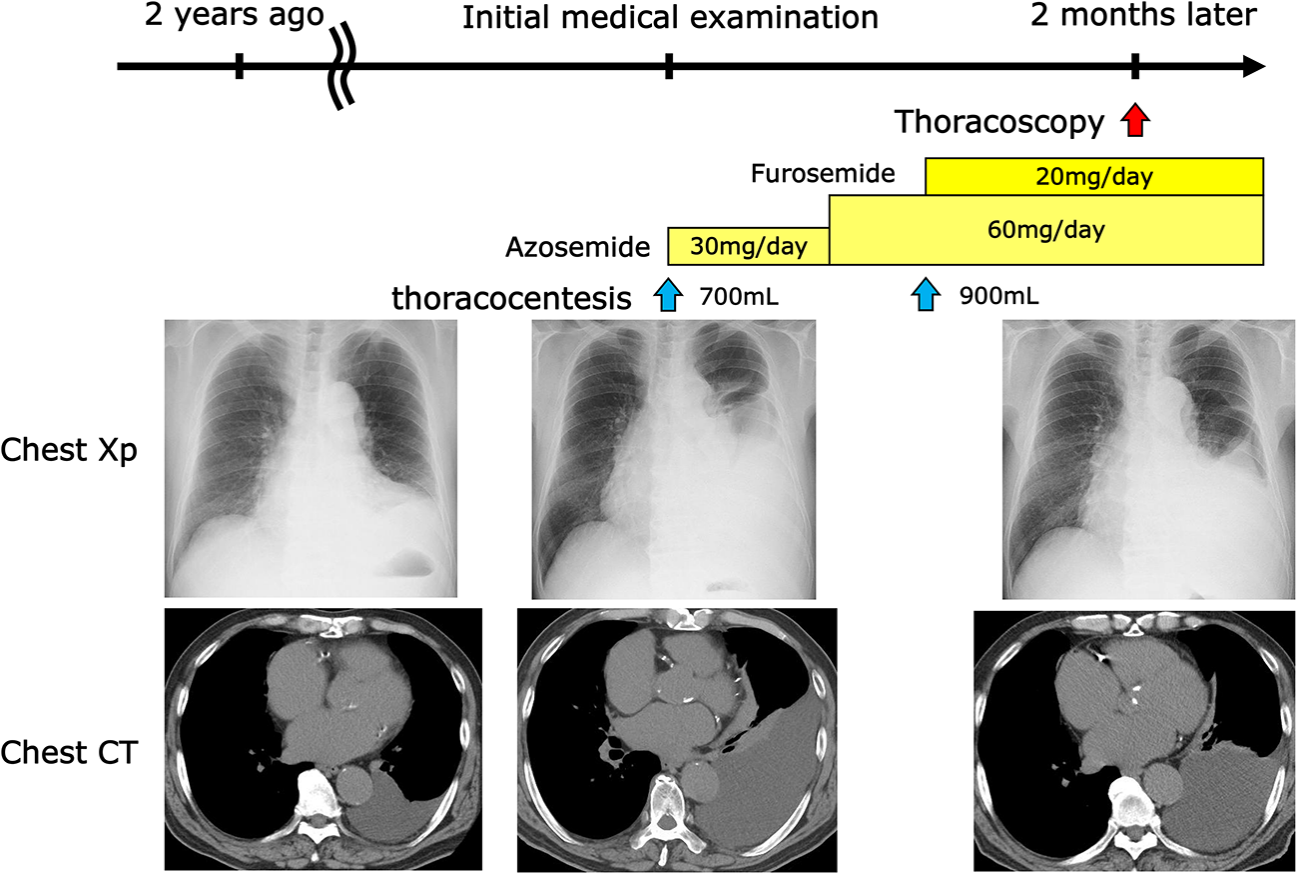
Left pleural effusion was noted on computed tomography (CT) scan approximately 2 years ago, but it was of a small volume and was under observation. A chest x‐ray revealed a significant increase in left pleural fluid, leading to a referral to our department. Although oral diuretics were initiated for treatment, but the left pleural effusion did not diminish. Subsequently, we conducted thoracoscopy for a definitive diagnosis.

**FIGURE 2:**
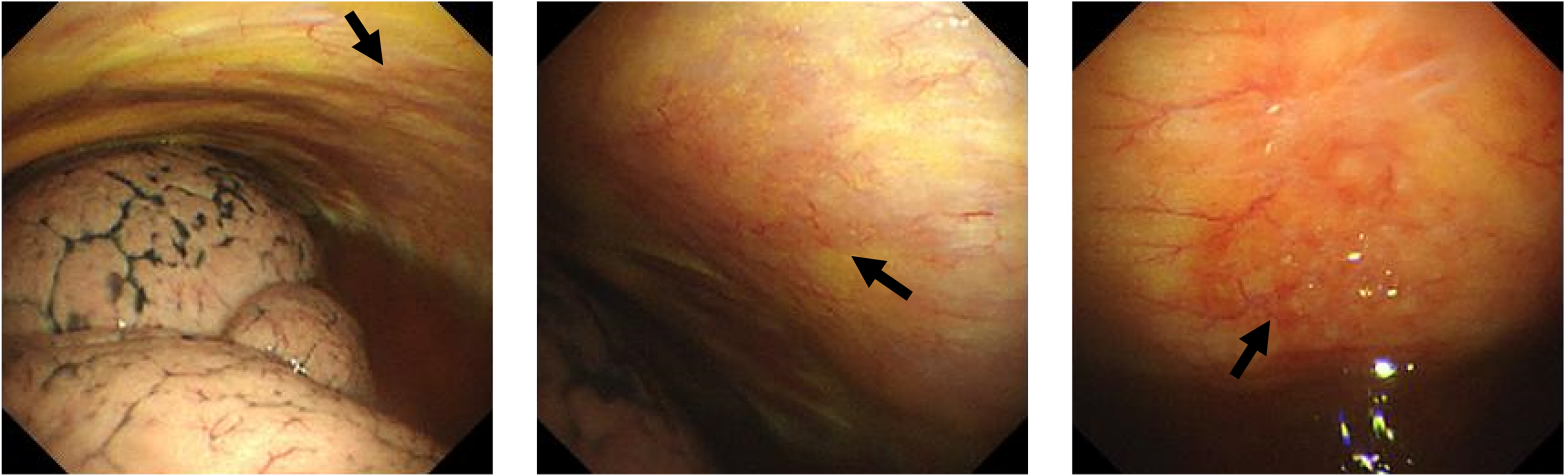
During thoracoscopy, smooth‐surfaced, indistinct‐bordered, erythematous lesion was observed on the left posterior wall (indicated by arrows).

The left pleural effusion initially presented as transudative, suggesting congestive heart failure. Oral diuretics were administered but failed to alleviate the left pleural effusion. Elevated serum IgG4, a Class III pleural effusion cytology, and positive Enzyme‐Linked Immunospot (ELISPOT) for tuberculosis in blood tests prompted a differential diagnosis encompassing tuberculous pleuritis, IgG4‐related pleuritis, and carcinomatous pleuritis. Consequently, a thoracoscopic pleural biopsy was performed under local anaesthesia 2 months after the initial examination (see Figure 1). During thoracoscopy, an erythematous lesion with a smooth surface and indistinct border was identified on the left posterior wall. A forceps biopsy was conducted at two locations (see Figure 2).

**FIGURE 3:**
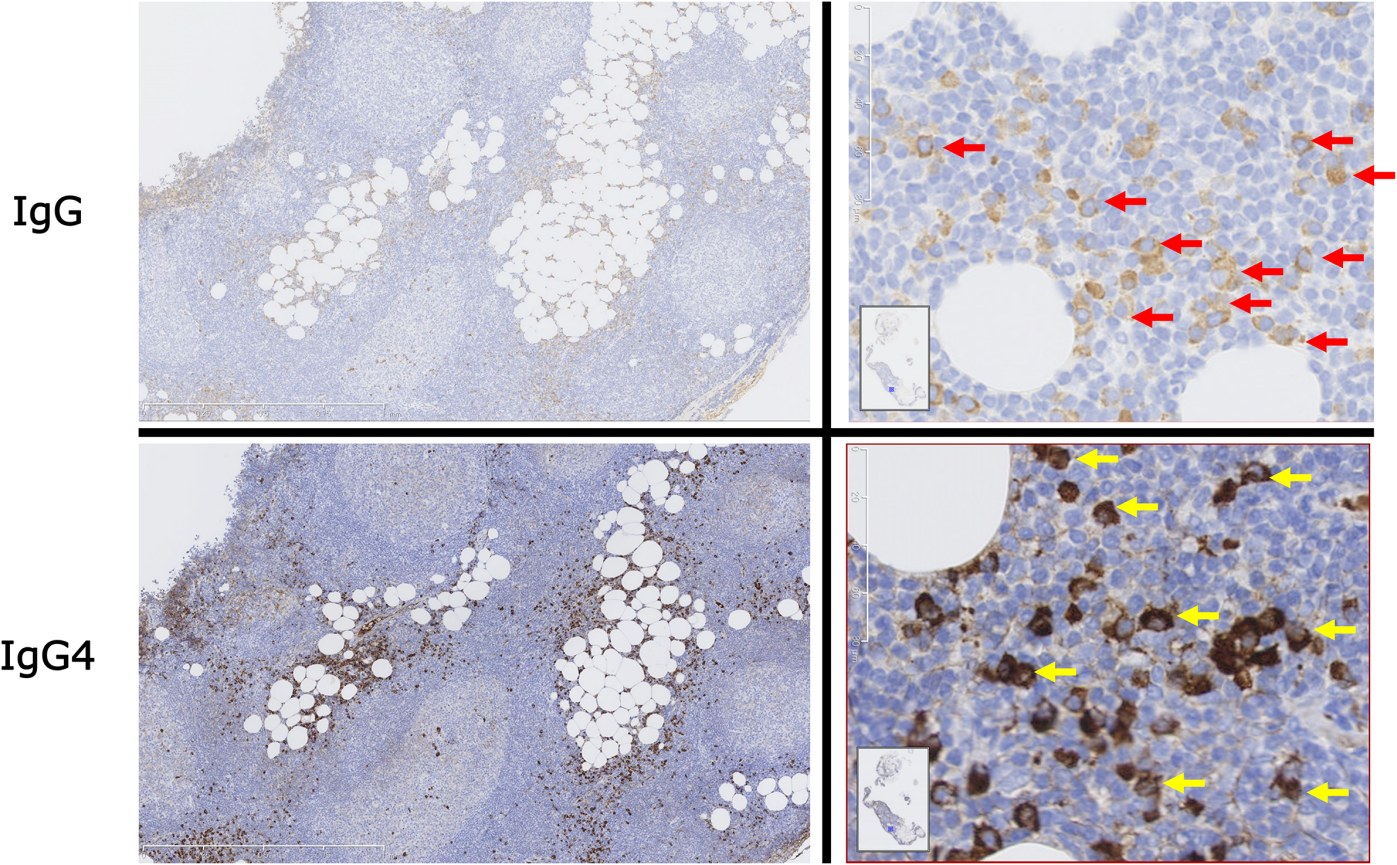
Biopsy specimens indicated hyperplasia of lymphatic follicles in the fatty tissue just below the mesothelium, with infiltration of plasma cells between the follicles. More than 50% of IgG‐positive plasma cells (indicated by red arrows) between follicles were IgG4‐positive, and the count of IgG4‐positive plasma cells (indicated by yellow arrows) exceeded 100 per high power field (HPF).

Pathological examination revealed hyperplasia of lymphatic follicles in the fatty tissue just below the mesothelium, along with plasma cell infiltration between the follicles. Calretinin‐positive pleural epithelium exhibited Ki‐67 < 1%, indicating low proliferative activity, and no definitive malignant findings or granulomas were observed. Polymerase chain reaction for Mycobacterium tuberculosis (TB‐PCR) yielded negative results, and Mycobacterium tuberculosis was not detected in the biopsy specimens. More than 50% of IgG‐positive plasma cells between follicles were IgG4‐positive, with the count surpassing 100 per high‐power field (HPF). Based on these findings, malignant pleural mesothelioma and tuberculous pleuritis were excluded, leading to the diagnosis of pleuritis associated with IgG4‐related disease (see Figure 3). Subsequently, prednisolone treatment was initiated due to an increase in the left pleural effusion. Following the commencement of steroid therapy, a notable reduction in the left pleural effusion was observed.

## DISCUSSION

This case report highlights transudative pleural effusions resistant to diuretic treatment, ultimately diagnosed as IgG4 related pleuritis through thoracoscopy under local anaesthesia, underscoring the significance of diagnostic scrutiny for transudative pleural effusions refractory to specific treatments. IgG4‐RD is a chronic, systemic fibroinflammatory disorder characterized by lymphoplasmacytic infiltration densely containing IgG4‐positive plasma cells, storiform fibrosis, and obliterative phlebitis, alongside elevated serum IgG4 levels.^1^ Approximately 40% of IgG4‐RD patients manifest thoracic lesions, with IgG4‐RD pleural diseases and effusions observed in 16% and 4.6%, respectively.^2^ In almost all cases, pleural effusions were exudative.^3^ The cause of transudative pleural effusions in our case remained unclear. Transudative pleural effusions develop whenever the hydrostatic and oncotic pressures across the pleural membrane are altered, such that the rate of fluid formation exceeds its rate of absorption. The endothelium of the pleural capillaries is intact and retains its normal sieving characteristics, so that cell and protein content in a transudative effusion is low.^4^ There were no findings of obliterative phlebitis in our biopsy specimens, but we hypothesize that obliterative phlebitis which is characteristic of IgG4‐RD may have led to extravascular leakage, resulting in transudative pleural effusions.

Given the positive ELISPOT for tuberculosis in our case without an elevation in pleural ADA, we conducted medical thoracoscopy under local anaesthesia for definitive diagnosis. Tuberculous pleuritis was considered negative since our case showed no evidence of Mycobacterium tuberculosis in pleural fluid culture or biopsy samples.

In conclusion, even transudative pleural effusion may result from inflammatory diseases. When unresponsive to treatment, a pleural biopsy (ideally thoracoscopic approach) should be considered for further investigation.

## AUTHOR CONTRIBUTIONS

The manuscript was prepared by YK under supervision of KF. Pleural biopsy was performed by YK, ST, YK and TF. Immunostaining was performed by HS. All authors read and approved the final manuscript.

## CONFLICT OF INTEREST STATEMENT

None declared.

## ETHICS STATEMENT

The Ethics Committee at Koka Hospital waived the need for approval. Written informed consent was obtained from the patient and his family to publish this article, in accordance with the journal's patient consent policy.

## Data Availability

The authors declare that the data supporting the findings of this case report are available within the article.
